# Grainyhead-Like 3 Influences Migration and Invasion of Urothelial Carcinoma Cells

**DOI:** 10.3390/ijms22062959

**Published:** 2021-03-15

**Authors:** Felix Wezel, Johannes Lustig, Anca Azoitei, Junnan Liu, Sabine Meessen, Gregoire Najjar, Viktor Zehe, Philipp Faustmann, Friedemann Zengerling, Axel John, Thomas Martini, Christian Bolenz, Cagatay Günes

**Affiliations:** Department of Urology, University of Ulm, 89081 Ulm, Germany; felix.wezel@uniklinik-ulm.de (F.W.); johannes.lustig@uni-ulm.de (J.L.); anca.azoitei@uni-ulm.de (A.A.); liu.junnan88@outlook.com (J.L.); Sabine.Meessen@uniklinik-ulm.de (S.M.); gregoire.najjar@uniklinik-ulm.de (G.N.); viktor.zehe@uniklinik-ulm.de (V.Z.); philipp.faustmann@uni-ulm.de (P.F.); Friedemann.Zengerling@uniklinik-ulm.de (F.Z.); axel.john@uniklinik-ulm.de (A.J.); thomas.martini@uniklinik-ulm.de (T.M.); Christian.Bolenz@uniklinik-ulm.de (C.B.)

**Keywords:** bladder cancer, GRHL3, differentiation, invasion, organ culture

## Abstract

Invasive urothelial carcinomas of the bladder (UCB) characteristically show a loss of differentiation markers. The transcription factor *Grainyhead-like 3* (*GRHL3*) plays an important role in the development and differentiation of normal urothelium. The contribution to UCB progression is still elusive. Differential expression of GRHL3 was assessed in normal human urothelium and in non-invasive and invasive bladder cancer cell lines. The contribution of GRHL3 to cell proliferation, viability and invasion in UCB cell lines was determined by gain- and loss-of-function assays in vitro and in an organ culture model using de-epithelialized porcine bladders. GRHL3 expression was detectable in normal human urothelial cells and showed significantly higher mRNA and protein levels in well-differentiated, non-invasive RT4 urothelial carcinoma cells compared to moderately differentiated RT112 cells. GRHL3 expression was absent in anaplastic and invasive T24 cells. Ectopic de novo expression of GRHL3 in T24 cells significantly impaired their migration and invasion properties in vitro and in organ culture. Its downregulation improved the invasive capacity of RT4 cells. The results indicate that GRHL3 may play a role in progression and metastasis in UCB. In addition, this work demonstrates that de-epithelialized porcine bladder organ culture can be a useful, standardized tool to assess the invasive capacity of cancer cells.

## 1. Introduction

Recent advances in molecular characterization have identified several subtypes of urothelial carcinoma of the bladder (UCB), with implications for prognosis and response to therapy [1,2,3]. The differentiation state of bladder cancer cells is associated with the aggressiveness and invasiveness of UCB [4]. Basal/squamous-type UCBs are characterized by a loss of terminal urothelial differentiation markers and show a more aggressive phenotype with worse oncological outcomes compared to luminal subtypes expressing differentiation-associated markers, such as peroxisome proliferator-activated receptor gamma (PPARγ), forkhead box protein A1 (FOXA1) and cytokeratin 20 [1,2,5].

The transcription factor Grainyhead-like 3 (GRHL3) holds an important role in the establishment of a urothelial phenotype in the developing mouse bladder and is mainly expressed in luminal, terminally differentiated umbrella cells [6,7]. GRHL3 is involved in the development and terminal differentiation of urothelial tissue [7,8,9]. Impaired *GRHL3* gene expression led to defective barrier formation of the urothelium and downregulation of uroplakins. Moreover, uroplakin II was shown to be a direct target of GRHL3 [7]. In human urothelium, serial transcriptome analysis by gene arrays identified a group of transcription factors such as PPARγ, FOXA1, E74 Like ETS Transcription Factor 3 (ELF3) and GRHL3 which are upregulated to mediate terminal differentiation after induction of differentiation in vitro [8]. Directed differentiation of human embryonic stem cells (ESCs) and induced pluripotent stem cells (iPSCs) towards a urothelial phenotype led to upregulation of GRHL3 [9]. The role of GRHL3 has been investigated in various tumor types; however, its role as an oncogene or tumor suppressor is still controversial [10]. GRHL3 has been suggested as a potential and independent prognostic factor in diffuse large B-cell lymphomas. Patients with GRHL3-positive tumors showed significantly lower 5-year survival than patients without GRHL3 expression, indicating an oncogenic role of GRHL3 [11]. An oncogenic effect of GRHL3 was also indicated in studies using colorectal cell lines. Hereby, the knockdown of GRHL3 impaired cell proliferation and migration and induced cell cycle arrest and apoptosis in colorectal adenocarcinoma DLD1 cells and colon cancer HT29 cells [12]. Furthermore, GRHL3 has also been found to be upregulated in advanced and extensively pre-treated human small-cell lung cancers and advanced adenocarcinomas of the lung, indicating a role in chemotherapy-resistant phenotypes [13]. Zhao et al. showed an inverse correlation of GRHL3 and E-cadherin expression profiles in the breast cancer cell lines MCF-7, A-431 and MDA-MB-231. The authors could show that GRHL3 overexpression in these cell lines led to lower E-cadherin expression and significantly higher cell migration and invasion, while GRHL3 knockdown in the invasive MDA-MB-231 cell line resulted in increased E-cadherin expression and impaired cell migration and invasion [14].

Other evidence suggests a tumor suppressor function of GRHL3 in some solid tumors. Expression studies of breast cancer patients revealed high GRHL3 protein expression in early stage breast cancer which decreased with tumor progression. Higher GRHL3 expression levels were associated with longer survival [15]. Two further studies defined GRHL3 as a potent tumor suppressor in human and mouse squamous cell carcinoma (SCC) [16,17]. Darido et al. could demonstrate that short hairpin RNAs (shRNA)-mediated GRHL3 knockdown in the human keratinocyte cell line HaCaT resulted in markedly reduced phosphatase and tensin homolog (PTEN) levels and higher proliferation rates. The authors identified a highly conserved GRHL3 binding site in *PTEN* promoter and could prove PTEN as a critical downstream target of GRHL3. Moreover, GRHL3 and PTEN expression levels in human SCC specimens and SCC cell lines were reduced compared to adjacent epidermis or HaCaT cell line, respectively. On the other hand, *GRHL3* levels are regulated by miR-21 [17,18].

To date, the role of GRHL3 in bladder carcinogenesis is yet unclear. In this study, we investigated the impact of GRHL3 in the proliferation, migration and invasion of urothelial cells by gain- and loss-of-function assays in bladder cancer cell lines. Furthermore, we established a standardized organ culture model using de-epithelialized porcine bladder for organotypic invasion studies.

## 2. Results

### 2.1. GRHL3 Expression Is Downregulated in Bladder Cancer Cells

**Figure 1:**
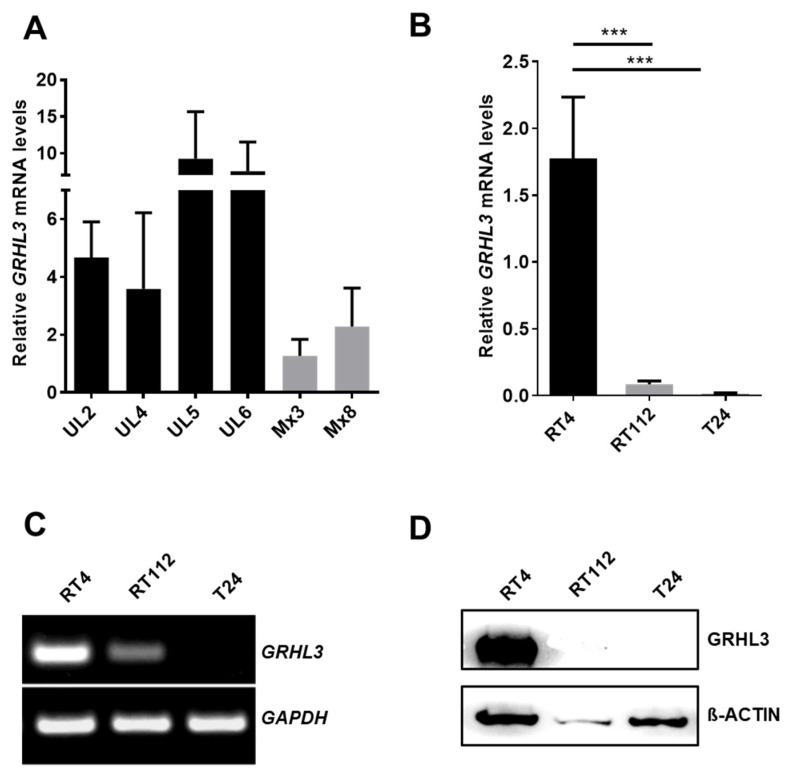
(**A**) Expression of Grainyhead-like 3 (*GRHL3*) transcript was readily detectable in freshly prepared normal human urothelial (NHU) epithelium and cultured primary urothelial epithelial cells (M × 3 and M × 8). (**B**–**D**) *GRHL3* expression was downregulated in invasive bladder cancer cell lines. (**B**) RT-qPCR and (**C**) semi-quantitative RT-PCR show lower expression of GRHL3 transcript in RT112 compared to non-invasive RT4 cells and absence of GRHL3 transcript in anaplastic T24 cells. *** indicates *p*-value < 0.001 (**D**) GRHL3 protein expression was only detected in RT4 cells by Western blot.

We firstly determined the expression of *GRHL3* mRNA levels in epithelial cells freshly prepared from normal human ureter tissue samples (UL2, UL4, UL5 and UL6) and in two cultured cell strains (Mx3 and Mx8) established from normal human ureter tissue (Figure 1A). As normal bladder urothelial tissue samples are difficult to obtain, we isolated total RNA from urothelial cells from the ureters of patients undergoing nephrectomy and determined *GRHL3* expression by real-time quantitative PCR. Expression of *GRHL3* mRNA was detected in the normal urothelium of four independent donors (Figure 1A). *GRHL3* mRNA levels were consistently lower in cultured urothelial cell strains when compared to freshly isolated, primary uncultured urothelium. In order to understand the contribution of *GRHL3* in bladder cancer cells, we next determined its mRNA levels in three bladder cancer cell lines by semi-quantitative RT-PCR. *GRHL3* mRNA levels were readily detectable in well-differentiated, low-grade, non-invasive RT4 cells (comparable to cultured normal human urothelial cells, Mx3 and Mx8). In contrast, GRHL3 expression was undetectable in the poorly differentiated, invasive bladder cancer cell line T24, assessed by RT-PCR (Figure 1B,C). Similarly, the GRHL3 protein was detectable in RT4 cells but not in T24 cells (Figure 1D). RT112 cells, an invasive cell line with limited potential for differentiation [19], expressed significantly lower *GRHL3* mRNA compared to RT4 cells but lacked protein expression (Figure 1B–D). Importantly, the absence of GRHL3 expression in T24 cells was not due to deletion of the *GRHL3* locus, as determined by genomic DNA PCR with two different regions of this gene (Supplementary Figure S1).

### 2.2. Ectopic GRHL3 Does Not Influence Cell Survival and Cell Cycle Profile of T24 Cells

**Figure 2:**
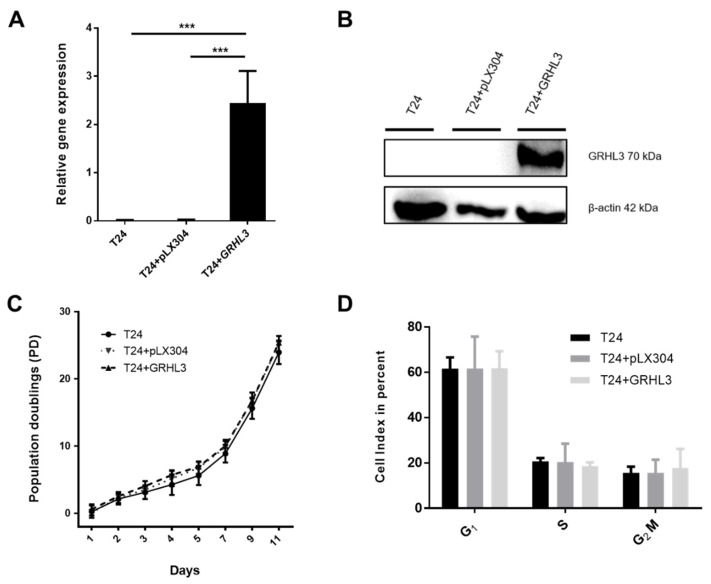
Ectopic expression of GRHL3 does not influence proliferation/survival rate and cell cycle profile of T24 cells (**A**,**B**) Forced stable expression of GRHL3 in T24 cells resulted in significantly higher transcript and protein expression levels compared to parental T24 cells and empty vector controls (pLX304). Three biological replicates were used for RT-qPCR experiments. *** indicates *p*-value < 0.001. (**C**) Calculated population doublings using Thiazolyl blue tetrazolium bromide (MTT) assays were comparable between GRHL3-overexpressing T24 cells, parental T24 cells and empty vector controls after 11 days in culture (T24 vs. T24 + pLX304 vs. T24+GRHL3: 23.92 vs. 25.19 vs. 25.40 population doublings (PD) at day 11; n.s). (**D**) Cell cycle analysis was performed by Propidium iodide (PI) staining and relative amounts of cells in the respective cell cycle phases are indicated. No difference could be observed in G1, S or G2/M phases between parental cells, empty vector controls and ectopic GRHL3-expressing cells (statistically n.s.).

Loss of GRHL3 expression may indicate a potential contribution of GRHL3 to bladder cancer progression. In order to assess its potential role in bladder cancer, we generated T24-GRHL3 (T24 + GRHL3) cells by ectopic stable expression using lentiviral vectors. Expression of GRHL3 was confirmed by RT-qPCR and Western blot (Figure 2A,B).

After establishing stable GRHL3 expression in T24 cells, we aimed to functionally assess its potential contribution to proliferation, migration and invasion in vitro. We could not observe any impact of GRHL3 overexpression on T24 cell survival and proliferation, assessed by population doublings (Figure 2C). This observation was in line with the cell cycle profiles as determined by flow cytometry (Figure 2D), indicating that GRHL3 did not influence the cell cycle profile in this setting.

### 2.3. Ectopic GRHL3 Impairs Migration Capacity of T24 Cells

**Figure 3:**
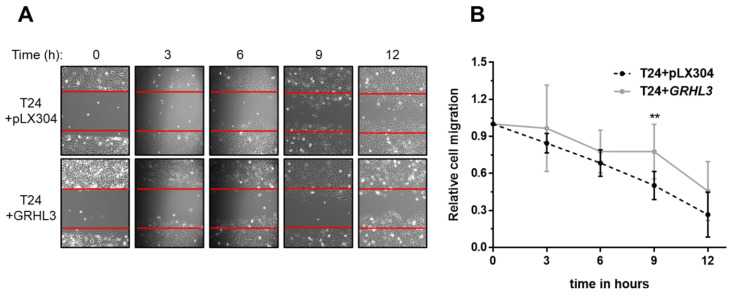
GRHL3 overexpression reduces migration in scratch wound assay. (**A**) Representative scratch wound experiment, displaying the original scratch wound gap in each picture by red lines during gap closure over 12 h. (**B**) Quantification of the “wound” closure shows that ectopic GRHL3-expressing T24 cells migrate significantly more slowly compared to empty vector controls (T24 + pLX304) at 6, 9 and 12 h (*p* = 0.0013 at 9 h). Seven replicates were performed in this experiment. ** indicates *p*-value < 0.01.

We used the established wound healing (or scratch) assay to evaluate the impact of GRHL3 on the migration capacity of T24 cells. GRHL3 overexpression resulted in significantly impaired migratory potential of T24 cells (T24+GRHL3) when compared to empty vector-transduced cells (T24 + pLX304) (Figure 3A,B).

### 2.4. Forced Ectopic GRHL3 Expression Impairs Invasion Capacity of T24 Cells

**Figure 4:**
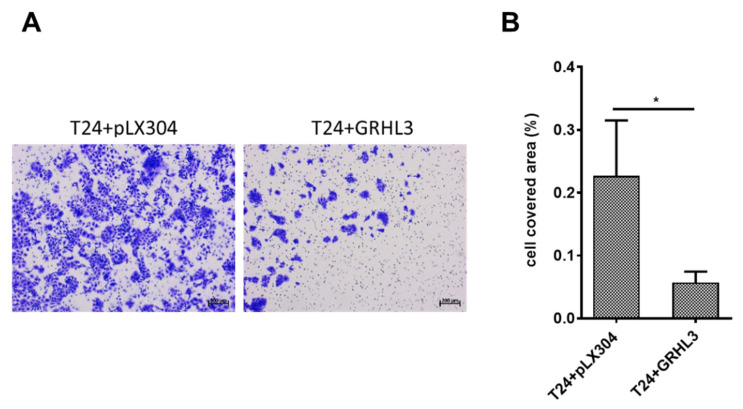
Ectopic overexpression of GRHL3 impairs invasion capacity of T24 cells in Boyden chamber assay. (**A**) Representative images from Boyden chamber assays showing “invaded” T24 cells stained with crystal violet, indicating lower invasive potential in GRHL3-overexpressing cells. Scale bar, 200 µm. (**B**) Analysis of the cell-covered area using software demonstrated that GRHL3-overexpressing cells (T24 + GRHL3) invaded through membranes to a significantly lower extent than empty vector controls (T24 + pLX304; *p* = 0.0317). SD bars are shown for a total of 70 images analyzed. * indicates *p*-value < 0.05.

Next, we investigated the impact of GRHL3 on the invasive capacity of T24 cells by two different experimental approaches. Invasion assessed by Boyden chamber experiments indicated that overexpression of GRHL3 significantly impaired the invasive capacity of T24 cells in comparison to empty vector controls (Figure 4A,B).

**Figure 5:**
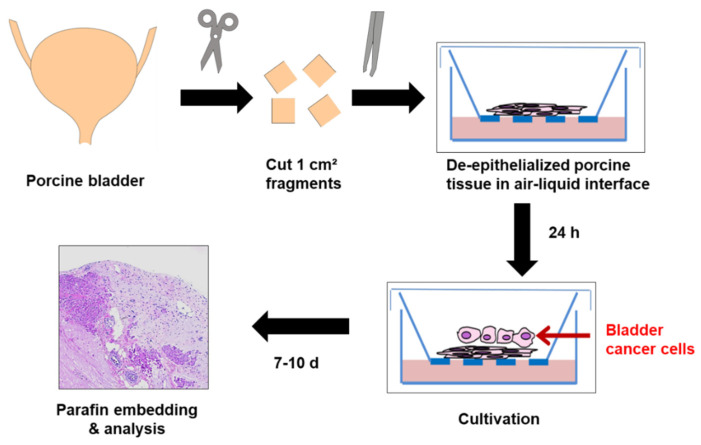
Schematic overview of ex vivo organ culture model for cell invasion. Porcine bladders were cut into 1-cm^2^ pieces and tissue was processed for de-epithelization. Cells were placed onto the de-epithelialized porcine bladder pieces and kept for 7–10 days in an air–liquid-interface before analysis.

To overcome limitations of the Boyden chamber as a purely in vitro assay for assessing invasion and migration of cultured cells, we established an organ culture model using de-epithelialized porcine bladders in an air–liquid interface, thus allowing organotypic cell–stroma interactions, which may potentially impact tumor cell invasion and progression [19,20] (Figure 5). Native or de-epithelialized porcine bladder tissues could be cultured for up to four weeks while maintaining their viability and morphological integrity. In de-epithelialized porcine bladders, we did not observe re-growth of potentially remaining urothelial cells after de-epithelialization, which we used as negative control.

**Figure 6:**
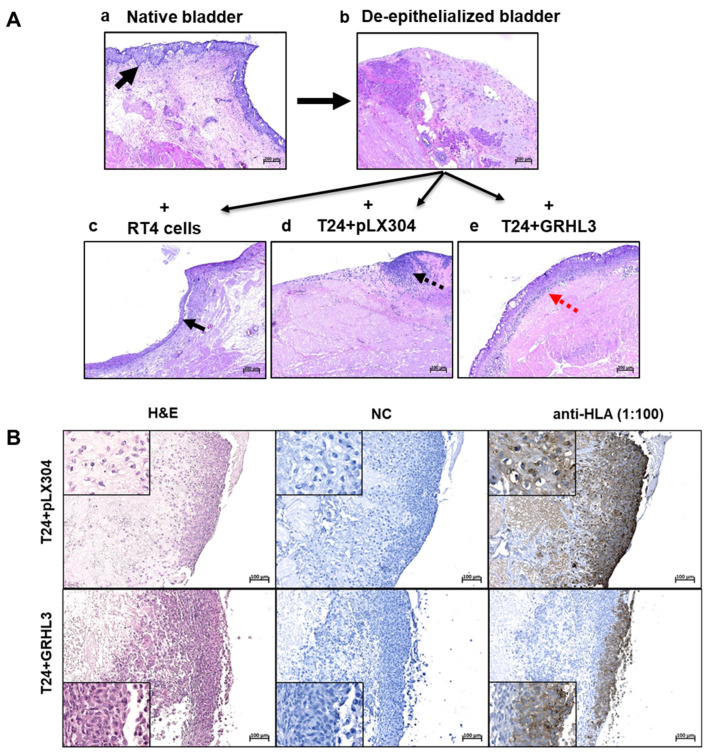
((**Aa**)–(**Ae**)) Representative formalin-fixed, paraffin-embedded (FFPE) hematoxylin and eosin (H&E) tissue sections of porcine bladders after 10 days in organ culture. (**Aa**) Native, untreated porcine bladder with intact morphology maintaining urothelium (black arrow), stroma and muscle tissue. (**Ab**) The de-epithelialized porcine bladder with stromal and muscle tissue, without urothelial re-growth, used as negative control. ((**Ac**)–(**Ae**)) Cell lines were seeded onto de-epithelialized bladder tissue and grown in organ culture. While non-invasive RT4 cells formed a stratified multi-layer on top of the basal membrane (**Ac**, black arrow), T24 cells with empty vector controls (T24 + pLX304) showed diffuse invasion into the stromal compartment (**Ad**, black dotted arrow). In contrast, T24 cells overexpressing GRHL3 (T24 + GRHL3) did not invade but instead formed a superficial multi-layer on the pig stroma, similar to non-invasive RT4 cells (**Ae**). Four biological replicates were performed for each experiment. Scale bar, 200 µm. (**B**) Anti-human leukocyte antigen (Anti-HLA) staining confirms the invasive capacity of T24 + pLX304 cells into the pig bladder stroma and muscle tissue as well as the reduced invasion ability upon overexpression of T24 + GRHL3. (H&E, hematoxylin and eosin; NC, negative control).

For invasion experiments, an incubation time in organ culture of between 7 and 10 days proved to be optimal and provided reproducible results. As controls, native and de-epithelialized porcine bladders were used (Figure 6(Aa,Ab)). Seeding non-invasive RT4 cells resulted in the formation of a stratified cell layer on top of the de-epithelialized basal membrane (Figure 6(Ac)). While the empty vector-transduced T24 cells (T24 + pLX304) invaded the stromal compartment, ectopic expression of GRHL3 reversed the invasive potential of T24 cells. GRHL3-transduced T24 cells instead formed a multi-layered epithelial lining (Figure 6(Ad,Ae)). These results not only support the findings obtained with the Boyden chamber assay but also highlight the importance of tumor–stroma interactions for establishing the tumor cell phenotype, as basically no invasion was observed in organ culture.

To validate these results and to exclude the possibility that re-growth of porcine cells may be responsible for epithelial cell formation, a human-specific anti-HLA antibody was used for immunolabeling (Figure 6B). Here, we could clearly identify the invading T24 cells carrying the empty vector within the porcine stroma, as well as the non-invading T24-GRHL3 cells on the surface of the porcine tissue, which were specifically labeled by anti-HLA antibody (Figure 6B). As controls for the anti-HLA antibody specificity, RT4 cells in the porcine organ culture system and untreated samples originating from human ureter and from porcine bladder were used (Supplementary Figure S2).

**Figure 7:**
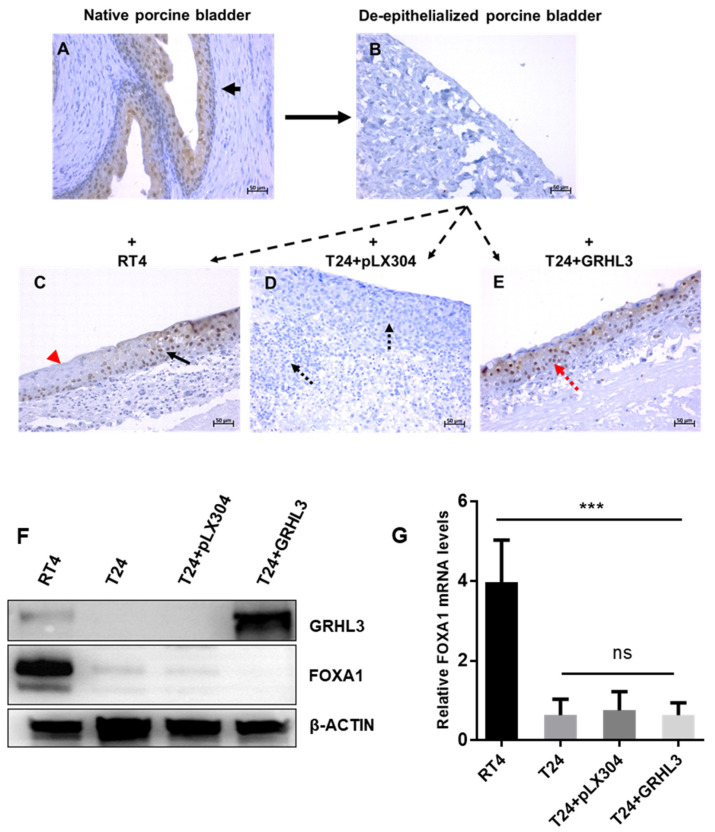
Ectopic expression of GRHL3 drives T24 cells towards a more differentiated phenotype when cultured in organ culture conditions allowing cell–stroma interactions. (**A**) Native porcine urothelium expresses FOXA1 in organ culture. (**B**) De-epithelialized porcine bladder was used as s negative control. (**C**) RT4 cells seeded on the de-epithelialized porcine bladder form a multi-layered epithelial lining. Not all cells are FOXA1-positive (red arrow), indicating partial differentiation. (**D**) T24+pLX304 cells invade into the porcine bladder stroma and muscle and lack FOXA1 expression (black dotted arrows). (**E**) GRHL3-expressing T24 cells do not invade and express FOXA1 (red dotted arrow). (**F**) Western blot shows FOXA1 protein expression only in RT4 but not in T24 cells, irrespective of ectopic GRHL3 expression when grown in 2D culture conditions. (**G**) Similarly, FOXA1 expression in 2D cell culture is not upregulated in T24 + GRHL3 cells. *** indicates *p*-value < 0.001; ns = not significant.

We next evaluated if the overexpression of GRHL3 drives T24 cells towards a more differentiated phenotype. Expression of FOXA1 was used as a differentiation marker by immunohistochemistry (IHC), Western blotting and RT-qPCR (Figure 7). FOXA1 expression was detectable in the epithelial cells of the untreated porcine bladder but not on the de-epithelialized porcine tissue by IHC (Figure 7A,B). As expected, FOXA1 expression in RT4 cells was detected but not in invasive empty vector-carrying T24 cells (Figure 7C,D). In contrast, GRHL3-expressing T24 cells (T24 + GRHL3) showed FOXA1 expression, similar to RT4 cells, supporting the idea that GRHL3 pushes T24 cells towards a more differentiated phenotype (Figure 7E). In order to demonstrate whether GRHL3 expression per se induces differentiation of T24 cells, we determined FOXA1 expression in 2D-cultured cells in vitro (Figure 7F,G). Interestingly, we did not observe FOXA1 upregulation under these conditions. RT-qPCR analyses of a set of uroplakins and PPARγ also did not show any upregulation of T24 + GRHL3 cells (Supplementary Figure S3), indicating that the GRHL3-induced differentiation of T24 cells required the stroma interaction in porcine organ culture. Although elucidation of the underlying molecular mechanisms was not the primary scope of this study, preliminary results indicate that GRHL3 overexpression leads to upregulation of the tumor suppressor protein PTEN, whose loss promotes invasiveness and progression in UCB by the subsequent activation of the Protein kinase B (PKB), also known as Akt, pathway [21,22] (Supplementary Figure S4).

### 2.5. GRLH3 Knockdown Increases Invasive Potential of RT4 in the Ex Vivo Organ Culture Model

**Figure 8:**
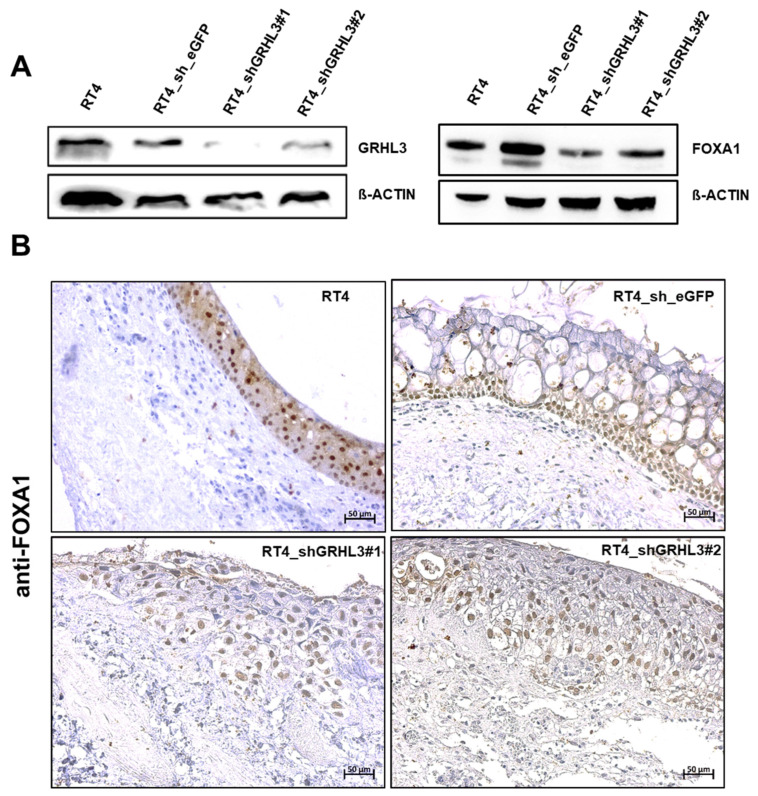
GRHL3 downregulation promotes a more invasive phenotype of RT4 cells in porcine organ culture. (**A**) Downregulation of GRHL3 protein by shRNAs in RT4 cell lines is shown by Western blot. Two shRNA constructs targeting GRHL3 and one control shRNA (sh_eGFP) were used to infect RT4 cells. Both shRNA constructs downregulated GRHL3 expression. FOXA1 expression is slightly reduced in RT4 cells after knockdown of GRHL3. (**B**) Immunolabeling of FOXA1 in RT4 cells grown in organ culture shows that FOXA1 expression can be detected in parental RT4 and in GRHL3 knockdown RT4 cells. Morphologically, parental and shRNA control-transduced RT4 cells formed a multi-layered epithelial lining, while knockdown of GRHL3 by both shRNAs showed more loose cell clusters with beginning stromal invasion (lower panel). Vacuoles in shRNA control-transduced RT4 cells may be indicative of cell stress. Scale bar, 50 µm.

To assess whether GRHL3 modulates the migratory and invasion capacities of urothelial cancer cells, we involved shRNA-mediated GRHL3 knockdown in the non-invasive RT4 cell line and performed functional experiments. The downregulation of GRHL3 with two different shRNAs was confirmed by Western blot. Western blot data also indicate that FOXA1 expression is slightly reduced after knockdown of GRHL3 in RT4 cells (Figure 8A). Again, invasion was assessed using the porcine organ culture model. Parental RT4 and RT4 cells transduced with control shRNA formed a multi-layered epithelial lining without signs of invasion. In contrast, RT4 cells transduced with GRHL3-shRNA grew in more loose cell clusters and we observed a substantial increase in the invasive capacity of RT4 cells. However, GRHL3 knockdown did not result in downregulation of FOXA1 expression (Figure 8B).

Scratch wound and Boyden chamber assays, however, did not increase the migratory or invasive capacity of RT4 cells upon GRHL3 knockdown (Supplementary Figure S5).

## 3. Discussion

UCB is amongst the most common cancers in the Western world and metastatic disease is associated with very poor survival rates. The state of differentiation has important implications for tumor biology, invasiveness and progression. UCB lacking markers of urothelial differentiation, i.e., basal and squamous-type tumors, shows the poorest prognosis [4]. The transcription factor GRHL3 is crucially involved in triggering urothelial differentiation during embryonic development and in inducing terminal-differentiated barrier-forming urothelium in vitro [7,8]. Thus, GRHL3 may be a surrogate marker of urothelial differentiation. The potential role of GRHL3 has been widely investigated in many different types of human tissues and cancer; however, contradictory roles of GRHL3 have been reported. It may promote cancer development or function as a tumor suppressor factor depending on the cancer type [23]. The functional role of GRHL3 in bladder cancer has been elusive.

The data presented in this study indicate a role of GRHL3 in the process of invasion during bladder cancer progression. Consistent with previous findings suggesting GRHL3 as a key regulator in urothelial differentiation, this study shows that GRHL3 is highly expressed in differentiated epithelial cells of normal human urothelium and well-differentiated RT4 bladder cancer cells, but its expression is reduced in RT112 cells and lost in the anaplastic bladder cancer cell line T24. Importantly, the study reveals that GRHL3 impairs migration and invasion of cancer cells, as ectopic expression of GRHL3 resulted in reduced migration and invasion capacities of T24 cells. Hereby, GRHL3-dependent changes in proliferation were not observed in vitro. Of note, loss of GRHL3 expression was not due to chromosomal deletion as normal human urothelial (NHU as well as RT4 and T24 cells contain the *GRHL3* genomic regions (Supplementary Figure S1).

Yet, the underlying mechanisms of *GRHL3* downregulation in T24 cells remain to be identified, i.e., genetic or epigenetic. Darido et al. described an miR-21-dependent proto-oncogenic network targeting GRHL3 as a tumor suppressor in the squamous cell cancer (SCC) HaCat cell line and SCC specimens [17]. Knockdown of GRHL3 by shRNA in HaCaT cells resulted in markedly reduced PTEN levels and higher proliferation rates. By identification of a highly conserved binding site in the PTEN promoter, PTEN was identified as a critical downstream target of GRHL3 [16,17]. Moreover, GRHL3 and PTEN expression in human SCC specimens was reduced compared to adjacent epidermis. In subsequent miRNA analyses, elevated levels of miR-21 were observed in SCC specimens and cell lines, indicating an oncogenic effect of miR-21 in the development of SCC [17]. Further investigations proved a direct transcriptional inhibitory regulation of GRHL3 on the miR-21 promoter in mouse and human SCC [18]. Similar mechanisms may be responsible in UCB and warrant further investigation. GRHL3 induces human epithelial tumor cell migration and invasion via downregulation of E-cadherin and modulation of the epithelial–mesenchymal transition (EMT) pathway [10,14]. In bladder cancer cell lines with de novo expression or knockdown of GRHL3, we have not observed differences in E-cadherin expression (not shown). Our preliminary data suggest that ectopic expression of GRHL3 may upregulate the tumor suppresser gene PTEN with subsequent AKT phosphorylation. Inactivation or downregulation of PTEN is commonly seen in many cancers, including UCB [21], as PTEN regulates and opposes the oncogenic Phosphoinositide 3-kinase (PI3K)-Akt pathway, i.e., by dephosphorylation [22].

Our study suggests GRHL3 as a potential prognostic marker in UCB. In silico analysis of the Cancer Genome Atlas (TCGA) data including 412 chemotherapy-naive, invasive, high-grade urothelial tumors [3] using the Human Protein Atlas platform (http://www.proteinatlas.org, accessed on 6 March 2021) showed a trend towards longer survival in UCB with high expression of GRHL3; however, it did not reach statistical significance (Supplementary Figure S6) [24]. Mutations in muscle-invasive bladder cancer are rather rare and account for only about 3% in the TCGA cohort, analyzed by cBioPortal (https://www.cbioportal.org, accessed on 6 March 2021). Further studies are required to assess the clinical utility of GRHL3 in UCB diagnosis and prognosis.

Our data show that NHU cells and well-differentiated, non-invasive RT4 cells strongly expressed GRHL3 at the transcript and protein levels. Knockdown of GRHL3 in RT4 cells by shRNA led to changes in cell morphology. RT4 shGRHL3 cells did not form a multi-layered epithelial lining but instead showed early signs of invasion, although this is difficult to quantify in organ culture. In vitro, however, wound healing and Boyden chamber assays showed no enhancement of invasion and migration of RT4 cells by GRHL3 downregulation. As a limitation, the remaining GRHL3 protein expression in RT4 cell lines has to be considered for the interpretation of the obtained results. Western blot experiments for FOXA1 show that knockdown of GRLH3 results in a partial but not a complete downregulation of FOXA1 (Figure 8A). The remaining FOXA1 signal may, therefore, be equally detectable in control and shRNA knockdown cells by IHC analysis in organ culture experiments. A complete knockout by, i.e., CRISPR/Cas9 could yield more pronounced effects.

GRHL3 has a crucial role in the differentiation of normal urothelium. Bell et al. suggested that PPARγ and GRHL3 participate in a Kruppel Like Factor 5 (KLF5)-dependent transcriptional network regulating urothelial differentiation [6]. Böck et al. have shown that GRHL3 lies downstream of PPARγ and ELF3, as PPARγ activation upregulates ELF3, which, again, regulates GRHL3 expression. ELF3 knockdown in normal urothelial cells led to reduced expression of FOXA1 and GRHL3 transcription factors in response to PPARγ activation [8]. Here, we also assessed the regulatory urothelial differentiation network by marker expression in T24 cells after ectopic overexpression of GRHL3. There was no upregulation of differentiation-associated markers FOXA1, PPARγ and the uroplakins 1a, 1b, 2, 3a and 3b in vitro (Supplementary Figure S3). Although induction of differentiation may not be expected in anaplastic cancer cells, we found de novo expression of FOXA1 by IHC analysis in GRHL3-expressing T24 cells when incubated in organ culture. These intriguing findings, together with an almost complete reversal of the invasive capacity of T24 cells upon GRHL3 overexpression, underline the crucial role of stromal tissue in the process of cancer progression and the relevance of organotypic conditions, crucially influencing cell behavior compared to purely in vitro experiments. These findings also suggest that GRHL3 alone is not sufficient to induce a urothelial differentiation program in anaplastic cancer cells. Moreover, the precise mechanisms behind the regulation of invasion/migration by GRHL3 remain largely elusive and may imply other signaling pathways, i.e., epithelial–mesenchymal transition (EMT) [25].

Organ culture models offer interaction with (organotypic) stroma and the extracellular matrix to more closely mimic the in vivo situation. Although they require a longer set-up compared to in vitro assays, they can potentially spare animal models for specific research questions. Booth et al. used human de-epithelialized urinary tract stroma in an air– liquid interface and could show that UCB cell lines could be kept in organ culture for up to eight weeks [19]. Specific cell characteristics, such as epithelial stratification or invasiveness, were highly reproducible. We adapted the model described by Booth et al. but chose porcine bladders for our model because the tissue is easier to obtain (abattoir waste) and porcine bladders are morphologically similar to normal human bladders with respect to structure, cell phenotype, marker expression and cell cycle control [26]. Although the use of human bladders may have species-associated advantages, porcine tissue may have less variability regarding the genetic (germ line variants) and epigenetic background (i.e., smoking, etc.). Thus, this model provides a very standardized setting. Similarly, rat organ culture bladder models were described before as a useful tool for invasion studies. However, this approach requires harvesting bladders from lab animals, and their handling is more difficult because of the size. Moreover, the phenotype of rat bladders can be significantly different to humans [27]. In such organ culture models, however, it is likely that multiple factors are regulating cell–cell communication, i.e., by paracrine or cell-bound signaling. The factors responsible for the distinct cell behavior of bladder cancer cells in organ culture and standard cell culture in our study remain unknown and are subject to future investigations.

In summary, our study shows convincing experimental evidence for an involvement of GRHL3 in bladder cancer cell invasion and differentiation, acting as a potential tumor suppressor protein. Further studies are required to assess GRHL3 as a diagnostic or prognostic biomarker in UCB. In addition, our study also shows that the porcine bladder organ culture model is a useful tool for studying cell invasion, in addition to standard 2D cell culture experiments, or could be suitable for drug testing in an organotypic culture model allowing cell–stroma interactions.

## 4. Materials and Methods

### 4.1. Cells

The human urothelial cell lines RT4 and T24 and HEK-293T cells were purchased from the American Type Culture Collection (ATCC^®^ HTB-2™, ATCC^®^ HTB-4™ and ATCC^®^ CRL-1573™, respectively). RT4 and T24 cells were kept in Roswell Park Memorial Institute (RPMI) 1640 medium and HEK-293T cells were kept in Dulbecco’s Modified Eagle Medium (DMEM) supplemented with 10% fetal calf serum (FCS), 1% penicillin/streptomycin (P/S) under standard culture conditions (ThermoFisher Scientific, Waltham, MA, USA). Detailed methods for the establishment and culture of normal human urothelial (NHU) cells have been described elsewhere [26,28]. Briefly, the isolated urothelium was incubated in collagenase and dispersed to a single cell suspension. Cells were seeded into Primaria™ plasticware in complete Keratinocyte Serum-Free Medium (KSFMc) containing recombinant epidermal growth factor and bovine pituitary extract (ThermoFisher Scientific, Waltham, MA, USA). Tissues were obtained at surgery from patients without urothelial malignancies with informed written consent and local research ethics committee approval (239/18). Two established normal human urothelial cell cultures derived from ureteric urothelium were provided by the Dept. of Urology, University Medical Center Mannheim, Germany.

### 4.2. Ectopic Expression of GRHL3 T24 Cells

T24 cells were infected with lentiviral particles for stable, ectopic expression of GRHL3 in T24. The empty vector (pLX304) was acquired from Addgene and a 1688-bp PCR product of GRHL3 cDNA was cloned by Nhel and Xbal (New England Biolabs, Ipswich, MA, USA) into the pLX304 backbone. HEK293T cells were then transfected with the psPAX2 and pMD2.G vector for generation of viral particles along with pLX304 or pLX304-GRHL3. T24 cells were infected and were kept under Blasticidin S (Sigma-Aldrich Chemie GmbH, Munich, Germany) selection (10 µg/mL).

### 4.3. shRNAs and Transfection in RT4 Cells

Samples of bacterial glycerol stocks of two shGRHL3 plasmids and one shGFP control plasmid (Table 1) of MISSION^®^ pLKO.1-puro System (Sigma-Aldrich, St. Louis, MO, USA) were placed overnight into shaking LB broth Miller (Cat No.: L2542, Sigma-Aldrich, St. Louis, MO, USA) medium containing 1% ampicillin. DNA was purified according to the supplier’s protocol (Cat No.:74044.50, Macherey-Nagel, Düren, Germany) and measured with an Epoch microplate spectral photometer. Embryonic kidney HEKHEK293T cells were then transfected with the psPAX2 and pMD2.G vector for generation of viral particles along with pLKO.1-puro anti-eGFP or pLKO.1-puro anti-GRHL3. RT4 cells were infected and were kept under puromycin (Sigma-Aldrich Chemie GmbH, Munich, Germany) selection (2 µg/mL).

### 4.4. PCR Primer

The primer sets used for semi-quantitative and real-time quantitative PCR (are presented in Table 2).

### 4.5. Western Blots

Western blot experiments were performed using 30 µg total cell lysates, as described [29]. Primary antibodies (Table 3) were incubated at 4 °C for 12 h. Secondary antibodies were incubated at ambient temperature for 2 h. Protein expression was measured using the “FusionFix” fluorescence and chemiluminescence imaging system (Vilbert Lourmat, Collégien, France). Further analyses were performed using ImageJ 1.51r.

### 4.6. Extraction of Genomic DNA

The genomic DNA was purified by using the QIAamp^®^ DNA mini kit according to the manufacturer’s recommendations (Cat No.: 51306, Qiagen, Hilden, Germany).

### 4.7. Extraction of Total RNA

Total RNA was extracted using the Qiagen RNeasy^®^ mini kit according to the supplier’s protocol (Cat No.: 74106, Qiagen, Hilden, Germany). Concentration and purity were measured with an Epoch microplate spectral photometer.

### 4.8. Reverse Transcription (RT)

Briefly, 100 ng total RNA in combination with 1 µL random primer and 1 µL oligo-dT was used for reverse transcription with the GoScript^TM^ reverse transcription system according the company’s protocol (Cat No.: A2801, Promega Corporation, Madison, WI, USA).

### 4.9. Polymerase Chain Reaction (PCR) and Real-Time

Semi-quantitative PCR for genomic DNA amplification was performed in a 25-µL reaction using standard conditions with the GoTaq^®^ G2 Flexi DNA polymerase system (Cat No.: M7808, Promega Corporation, Madison, WI, USA) with 100 ng genomic DNA using the following conditions: 1 × (95 °C, 2 min), 30x (95 °C, 30 s; 55 °C, 30 s; 72 °C 30 s) and 1 × (72 °C, 5 min).

### 4.10. Real-Time Quantitative PCR (RT-qPCR)

Real-time qPCR was performed as previously described [29] using the iTaq^TM^ Universal SYBR^®^ Green supermix (Cat No.: 1725120, Bio-Rad Laboratories Inc., Hercules, CA, USA) on a Viia7 q-RT-PCR device (ThermoFisher Scientific, Waltham, MA, USA). The ∆∆CT value was calculated to show the fold change of gene expression between two samples.

### 4.11. Population Doubling Assay

Cells were seeded in 24-well plates (1500 cells/well) and maintained in culture conditions up to 11 days and counted in improved Neubauer chambers. Cells were passaged after 5 days and 1500 cells were reseeded in new 24-well plates. Population doublings were calculated using the formula $PD=\log\frac{N}{N0}/\log2$.

### 4.12. Wound Healing Assay

The wound healing assay was performed using IBIDI 4-well culture inserts, according to the manufacturer’s protocol (Cat No.: 80469, ibidi Gmbh, Gräfelfing, Germany). Pictures were taken at different time points depending on the cell line with an Axiostar Vert A1 microscope and its AxioCam Cm1 camera (Zeiss, Oberkochen, Germany) with 10 times magnification. Pictures were analyzed with ImageJ 1.51 r software and its plugin, MRI_Wound_Healing_Tool.ijm.

### 4.13. Boyden Chamber Assay

The Boyden chamber assay was performed according to the manufacturer’s protocol (Corning Inc., Corning, NY, USA). Briefly, on day one, 80% confluent target cells were starved in serum-free media for 24 h in normal culture conditions. Following the manufacturer’s protocol, Falcon^®^ cell culture inserts with 8.0-µm pores (Corning Inc., Corning, NY, USA) were sealed with 100 µL Corning^®^ Matrigel^®^ matrix at a concentration of 3 mg/mL. The next day, 1 × 10^5^ starved cells were resuspended in 500 µL RPMI medium 1640 and seeded on the Matrigel^®^ matrix. The wells of the plate were filled with 500 µL complete RPMI medium as a chemoattractant for invasion. The cells were kept in culture conditions for 48 h to allow the invasion through the Matrigel^®^ matrix and the insert membrane. The invaded cells on the insert bottom were carefully washed in Dulbecco’s phosphate-buffered saline (DPBS) three times. For fixation, the cells were incubated in 500 µL of a 5% glutaraldehyde solution for 30 min. Then, cells were washed and visualized using 0.2% crystal violet solution.

### 4.14. Porcine Bladder Organ Culture Model

Pig bladders were kindly supplied by a local abattoir, which would otherwise have been discarded. Bladders were opened and washed in DPBS containing 3% penicillin/streptomycin three times. Then, small pieces of approximately 1 cm² were cut with surgical disposable scalpels and placed in 0.5% Dispase^®^ II (Cat No.: D4693, Sigma-Aldrich, St. Louis, MO, USA) overnight at 4 °C to loosen up urothelial cells from the base membrane. The de-epithelialized pieces were placed in 70-µm cell strainer inserts within a 6-well plate containing 6.5 mL optimized Waymouth medium supplemented with 10% FCS, 300 µg/mL L-ascorbic acid, 0.45 µg/mL ferrous sulphate heptahydrate and 2 µg/mL hydrocortisone (all from Sigma-Aldrich, St. Louis, MO, USA), as previously described [19]. The stromal tissue fragments were kept in culture conditions for 48 h to preclude potential bacterial or fungal contaminations. For cell seeding, 1 × 10^6^ cells were centrifuged in a Megafuge 1.0 R centrifuge. The pellet was resuspended in 5 µL complete Waymouth medium and then seeded on the de-epithelialized pig bladder. The exact amount of 6.5 mL was necessary to keep cells in air and tissue in medium. This air–liquid interface led to a diffusive nutrition and is elementary as a chemoattractive stimulus for invasion. The medium was changed on alternate days. After 7 days, the tissue was removed, fixed in 4% Paraformaldehyde (PFA) solution and embedded in paraffin in a semi-enclosed bench-top tissue processor (Leica, Wetzlar, Germany). For histological analysis, paraffin blocks were cut into 3-µm thick sections by a fully automated rotary microtome RM2035 (Leica, Wetzlar, Germany) and placed on SUPERFROST^®^ PLUS microscope slides for further analysis.

### 4.15. Data Processing, Statistical Analyses and Graphic Representations

Statistical analyses and graphic representations were performed using GraphPad Prism 6. Gene expression studies were analyzed by using Student’s *t*-test with a minimum of at least three replicates. The wound healing assay and the Boyden chamber assay were analyzed by multiple Student’s *t*-test.

## Figures and Tables

**Table 1:** Mission^®^ pLKO.1-puro anti-GRHL3 plasmids.

Plasmid	Plasmid ID	Cat No	Sequence in 5′-3′
shGRHL3#1	NM_021180.2-439s1c1	NM_021180.2	CCGGGCTCAAGAAGAATAACCTGATCTCGAGATCAGGTTATTCTTCTTGAGCTTTTT
shGRHL3#2	NM_021180.2-236s1c1	NM_021180.2	CCGGGCCTTGAGCTTCCTCTATGATCTCGAGATCATAGAGGAAGCTCAAGGCTTTTT
sheGFP	MISSION^®^ pLKO.1-puro eGFP shRNA Control	SHC005	CCGGTACAACAGCCACAACGTCTATCTCGAGATAGACGTTGTGGCTGTTGTATTTTT

**Table 2:** Primer sequences for RT-qPCR.

Gene	Forward Primer (5′-3′)	Reverse Primer (5′-3′)
Forkhead box protein A1 (FOXA1) (qPCR)	ACTGTGAAGATGGAAGGGCA	AGTAGGCCTCCCTGCTTGT
Glyceraldehyde 3-phosphate Dehydrogenase (GAPDH) (qPCR)	AGATCCCTCCAAAATCAAGTGG	AAAAGGGTCATCATCTCTGCC
Grainyhead Like Transcription Factor (GRHL3) (qPCR, RT-PCR)	ATTGACGTGGCTGACTGCAA	GCTCAGACAGTTTACGCCGA
Peroxisome proliferator-activated receptor gamma (PPARγ) (qPCR)	TATTCTCAGTGGAGACCGCC	AGGGCTTGTAGCAGGTTGTC
Phosphatase and tensin homolog (PTEN) (qPCR)	GGCACCGCATATTAAAACGTA	ATGCCATTTTTCCATTTCCA
Uroplakin 1a (UPK1a) (qPCR)	GTGGTGGGCCTGCTAGTTG	TATACACGCTACTGGTCGGCT
Uroplakin 1b (UPK1b) (qPCR)	CCAAAGACAACTCAACTGTTCGT	AATGCCGCAACAACCAATAATC
Uroplakin 2 (UPK2) (qPCR)	ACCAGGTGACAAACCTCGTG	TGTTCCTTCGAGGGAGTGTG
Uroplakin 3a (UPK3a) (qPCR)	CGTGGACATGGGGAGTTCTG	TCACGGACGTGTAGGAAGACT
Uroplakin 3b (UPK3b) (qPCR)	CCTCTACCATGCGCTTCTCC	ATGTGGTGGGTCATGTAGCG
GRHL3-3′-untranslated region (UTR) (RT-PCR)	CACACAACCTCTCCACATGC	TCAGGGAGCAGATTCAAGCA
GRHL3-coding (RT-PCR)	TCAACGGAAAAGCAGTGTGG	CGTTAGGCCGTGCTTCTTAC

**Table 3:** Antibodies used for Western blots.

Scheme	Antigen	Clone	Dilution	Provider
Mouse	GRHL3	C-12, sc-398838, IgG2b	1:1000	Santa Cruz, Dallas, TX, USA
Mouse	GRHL3	Aa529-578	1:500	Lifespan Biosciences, Seattle, WA, USA
Horse	Mouse IgG	HRP-linked, #7076	1:2000	Cell Signaling Technology, Danvers, MA, USA
Rabbit	Total Akt	#75692S (D9R8K)	1:2000	Cell Signaling Technology, Danvers, MA, USA
Rabbit	Phospho-Akt (Ser473)	#4060 (D9E)	1:2000	Cell Signaling Technology, Danvers, MA, USA
Rabbit	PTEN	#9559 (138G6)	1:1000	Cell Signaling Technology, Danvers, MA, USA
Chicken	Rabbit IgG	HRP-linked, sc-2955	1:2000	Santa Cruz, Dallas, TX, USA
Mouse	β-Actin	AC-15	1:10,000	Sigma-Aldrich, St. Louis, MO, USA

## Data Availability

Data is contained within the article or Supplementary Mmaterial.

## Supplementary Materials

The following are available online at https://www.mdpi.com/1422-0067/22/6/2959/s1.
