# Supplementary material for: Grainyhead-Like 3 Influences Migration and Invasion of Urothelial Carcinoma Cells
Source: Int J Mol Sci. 2021 Mar 15;22(6):2959. doi: 10.3390/ijms22062959 (PMC8000182; doi:10.3390/ijms22062959)
Supplement: Supplementary file 1 [file ijms-22-02959-s001.pdf]

Supplementary Materials:

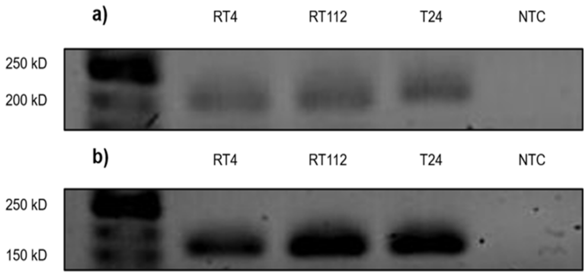

Supplementary Figure S1. Detection of genomic *GRHL3* DNA by genomic PCR. The GRHL3 gene is located on chromosome 1p36.11. Two primer pairs (*GRHL3* coding; *GRHL3*-3′ UTR) and genomic DNA were used for amplification of the gene. (**TOP**) The primer "*GRHL3* coding" amplified a 203-bp product containing introns and exons to include DNA and exclude all mRNA variants (Figure 7a). (**BOTTOM**) The second primer pair—*GRHL3*-3′ UTR—amplified a 156-bp product in the 3′ untranslated region (UTR). The results of both PCR reactions with different primer pairs indicate the presence of *GRHL3* gene region in RT4, RT112 and T24. NTC—negative template control.

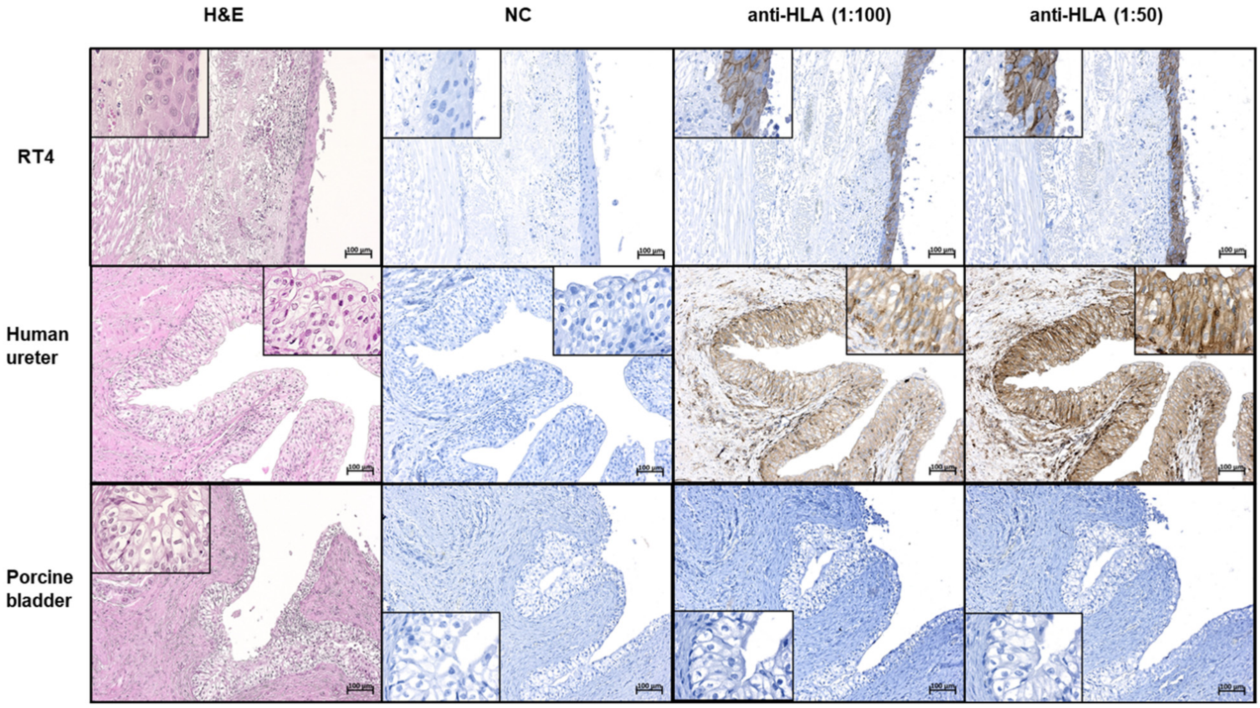

Supplementary Figure S2. Control experiments showing the specificity of the anti-HLA antibody. Human RT4 bladder cancer cells grown on de-epithelialized porcine bladder (upper panel) and normal human ureter (middle panel) immunoreact with anti-HLA antibody, while there is no immunoreactivity in porcine bladder (lower panels). (H&E, hematoxylin and eosin; NC, negative control).

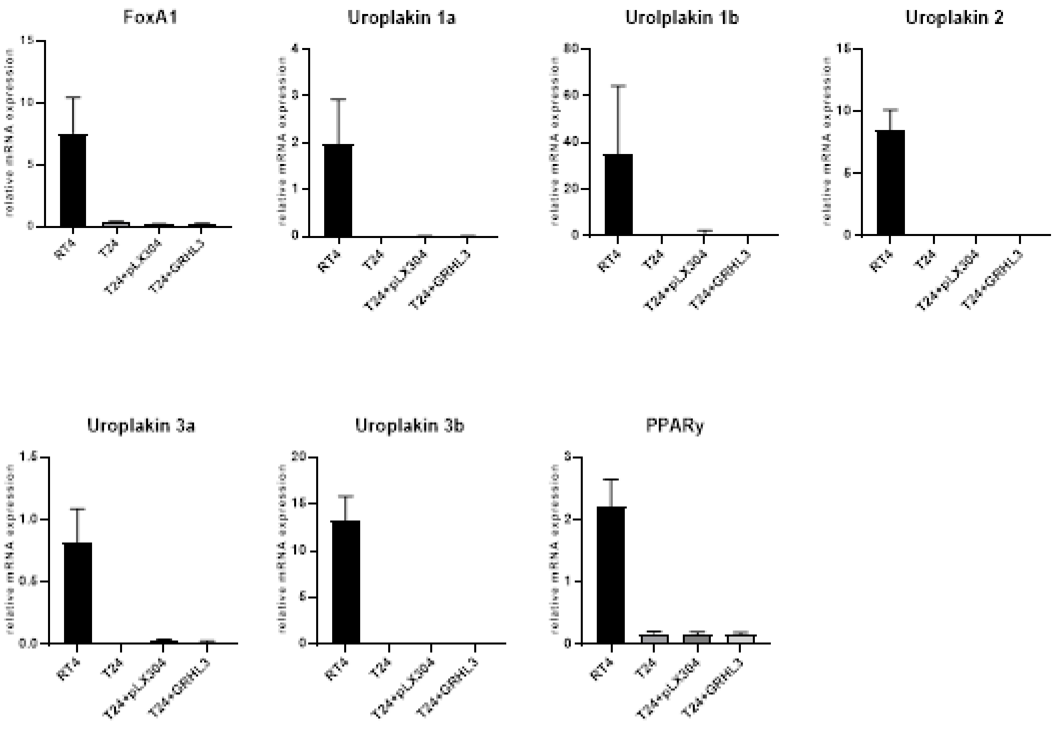

Supplementary Figure S3. RT-qPCR assessing urothelial differentiation-associated genes.

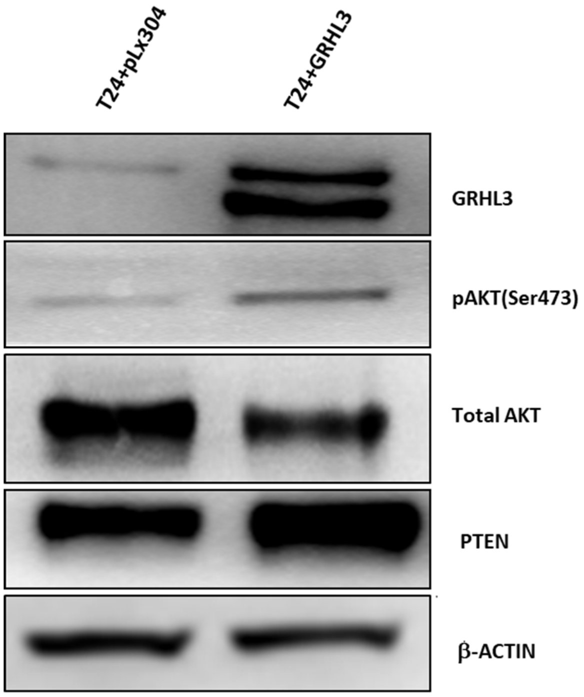

Supplementary Figure S4. Western blot shows a trend towards upregulation of PTEN and phosphorylated AKT (pAKTSer473) in T24 cells overexpressing GRHL3 (T24 + GRHL3) compared to empty vector controls (T24 + pLX304).

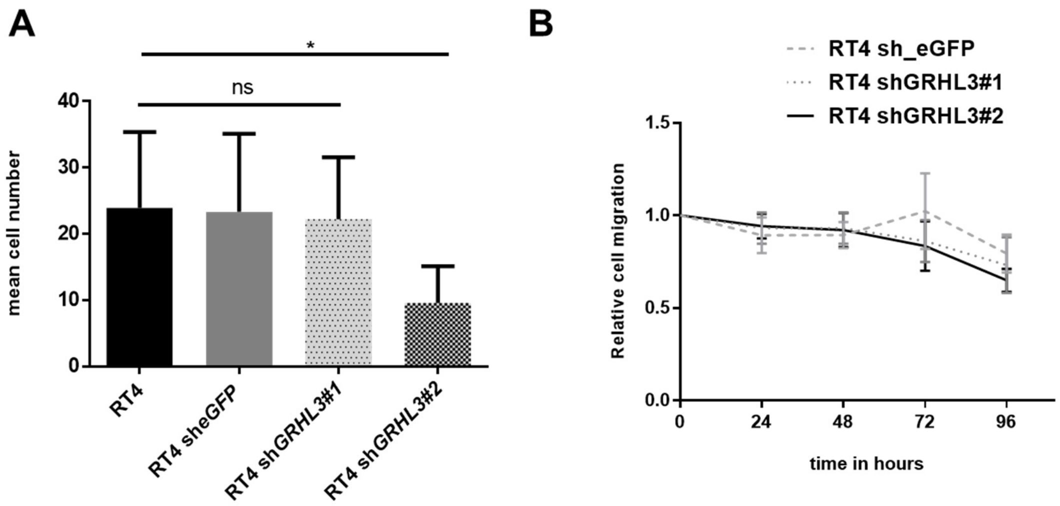

Supplementary Figure S5. (**A**) Boyden chamber assay demonstrates the very low invasiveness of RT4 cells *in vitro*. GRHL3 knockdown did not enhance invasiveness. (**B**) Wound healing assays showed no difference in migration between RT4 cells transduced with control vector (sh-eGFP) or GRHL3 knockdown (shGRHL3) (n.s. at all time points).

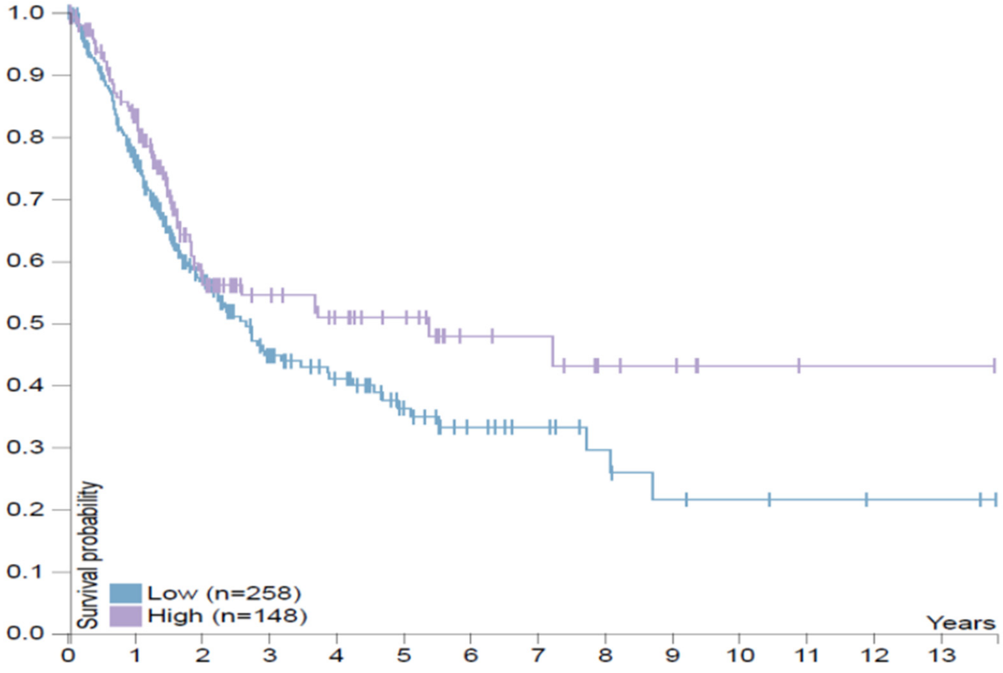

Supplementary Figure S6. Kaplan–Meier survival curves of The Cancer Genome Atlas (TCGA) bladder cancer cohort (3), discriminating between high vs. low expression of *GRHL3* (based on RNA sequencing). High *GRHL3* expression showed a trend towards longer survival, but differences were not statistically significant ($p = 0.074$). The cut-off values were calculated to yield the maximal difference with regard to survival between the two groups at the lowest log-rank P-value, selected on survival analysis. Source: humanproteinatlas.org.
